# Visceral fat, but not subcutaneous fat, is associated with lower core temperature during laparoscopic surgery

**DOI:** 10.1371/journal.pone.0218281

**Published:** 2019-06-12

**Authors:** Ryohei Miyazaki, Sumio Hoka, Ken Yamaura

**Affiliations:** 1 Operating Rooms, Kyushu University Hospital, Fukuoka, Japan; 2 International University of Health and Welfare, Fukuoka, Japan; 3 Department of Anesthesiology and Critical Care Medicine, Graduate School of Medical Sciences, Kyushu University, Fukuoka, Japan; University of Colorado, Anschutz Medical Campus, UNITED STATES

## Abstract

**Background:**

Previous studies suggest that lower BMI is a risk factor for intraoperative core hypothermia. Adipose tissue has a high insulation effect and is one of the major explanatory factors of core hypothermia. Accordingly, determining the respective influence of visceral and subcutaneous fat on changes in core temperature during laparoscopic surgery is of considerable interest.

**Methods:**

We performed a prospective study of 104 consecutive donors who underwent laparoscopic nephrectomy. Temperature data were collected from anesthesia records. Visceral and subcutaneous fat were calculated by computed tomography (CT) or ultrasound. For ultrasound measurements, preperitoneal fat thickness was used as an index of visceral fat. Multiple linear regression analysis was performed at 30, 60, and 120 minutes after the surgical incision to identify the predictive factors of body temperature change. The potential explanatory valuables were age, sex, BMI, visceral fat, and subcutaneous fat.

**Results:**

BMI (β = 0.010, 95%CI: 0.001–0.019, p = 0.033) and waist-to-hip ratio (β = 0.424, 95%CI: 0.065–0.782, p = 0.021) were associated with increased core temperature at 30 minutes after the surgical incision. Ultrasound measured-preperitoneal fat was significantly associated with increased core temperature at 30 and 60 minutes after the surgical incision (β = 0.012, 95%CI: 0.003–0.021, p = 0.009 and β = 0.013, 95%CI: 0.002–0.024, p = 0.026). CT-measured visceral fat was also associated with increased core temperature at 30 minutes after the surgical incision (β = 0.005, 95%CI: 0.000–0.010, p = 0.046). Conversely, subcutaneous fat was not associated with intraoperative core temperature. Male sex and younger age were associated with lower intraoperative core temperature.

**Conclusions:**

Visceral fat protects against core temperature decrease during laparoscopic donor nephrectomy.

## Introduction

Inadvertent hypothermia is a common intraoperative complication. Hypothermia can lead to adverse patient outcomes, including shivering, increased blood loss and transfusion[1,2], surgical site infection[3], and reduced clearance of various drugs[4]. These complications may lead to higher mortality rates and longer hospital stays[5].

Core hypothermia results from core-to-peripheral redistribution of body heat, and from heat loss via the skin to the environment[6]. It is difficult to prevent the initial intraoperative reduction in core temperature[6,7]. The reported predictive factors for intraoperative hypothermia include height, weight, body weight-to-body surface area ratio, ambient temperature, prewarming and vasopressors[8–12].

Adipose tissue has a high insulation effect, and one previous study reported that patients with excess fat exhibit relatively small core temperature decreases during surgery[13]. Although overall adiposity can be roughly indicated by BMI, BMI is simply calculated using height and weight, and does not accurately reflect the amount of adipose tissue. A more accurate method is to measure the amount of fat using computed tomography (CT) or ultrasound [14, 15].

Visceral fat and subcutaneous fat differ in the types of adipocytes, innervation ratios, and endocrine functions[16]. Anatomically, visceral adipose tissue is more vascular than subcutaneous adipose tissue, and visceral fat venous blood is drained through the portal vein[16]. We have supposed that visceral fat is less affected by ambient temperature than subcutaneous fat, but is more susceptible to the influence of pneumoperitoneum gas temperature during laparoscopic surgery. In the present study, we examined which type of fat, visceral or subcutaneous, had a greater influence on core temperature change during laparoscopic surgery. We evaluated visceral and subcutaneous fat using both ultrasound and CT to minimize the influence of fat distribution and interobserver differences on the measurements.

## Materials and methods

This clinical trial was approved by the Ethical Committee for Clinical Studies of the Kyushu University School of Medicine, and was prospectively registered at UMIN Clinical Trial Registry (UMIN000019276). One-hundred-and-twenty patients scheduled for donor nephrectomy were enrolled. All surgeries were performed through the transperitoneal approach in the left renal position. Written informed consent was obtained from each patient prior to participation in this study. Patients with cardiovascular disease, peripheral vascular disease, preoperative hypo- or hyperthermia, an age of 70 years or older, American Society of Anesthesiologists Physical Status classification (ASA-PS) > 2, autonomic disorder, or thyroid disease were excluded. Patients undergoing emergency surgery were also excluded.

### Induction and maintenance of anesthesia

None of the study participants were premedicated. Electrocardiography, pulse rate, blood pressure, respiratory rate, and oxygen saturation were constantly monitored from the time of each patient’s arrival in the operating room. All patients underwent general anesthesia. No patient was given an epidural anesthesia. After arrival in the operating room, the patients were preoxygenated with 100% oxygen via a facemask. Anesthesia was induced with propofol (1.5 mg/kg) and fentanyl (1.5 μg/kg). Rocuronium (0.6 mg/kg) was administered to facilitate tracheal intubation. After the induction of anesthesia, a temperature probe (Smiths Medical, London, UK) was inserted into the distal esophagus to measure the core body temperature, as previous reports have shown that esophageal temperature is a reliable measure of core temperature [17,18]. Anesthesia was maintained with desflurane (4.0–6.0% in oxygen and air), and patients received an infusion of remifentanil at a rate of 0.2–0.3 μg/kg^/^min. Remifentanil and rocuronium consumptions were calculated using the ideal body weight. Anesthetic, hemodynamic, and fluid management were at the discretion of the attending anesthesiologist.

### Temperature management

Body temperature was measured in the ward 1 hour before surgery as a baseline temperature to exclude the presence of preoperative hypo- or hyperthermia. Patients did not receive prewarming. A forced-air warming device with an underbody disposable blanket (3M Bair Hugger Model 635) was used to prevent hypothermia. Body warming by the forced-air warmer was started at the time of the surgical incision; the set temperature was 38°C. The environmental temperature in the operating room was set at 25°C with 40% relative humidity until the end of surgery. Intravenous fluids were warmed to 42°C via a fluid warmer (HOTLINE, Smiths Medical, Rockland, MA). Temperature data were recorded every minute, and downloaded as a digital file.

### Measurement of adipose tissue

Anthropometric tape was used to measure the waist circumference at the level of the umbilicus and the maximum hip circumference during the end-expiratory phase in the standing position. Each measurement was repeated three times, and the average of the three measurements was used in subsequent analyses. The waist-to-hip ratio (WHR) was calculated and used as index of central obesity [19].

**Fig 1:**
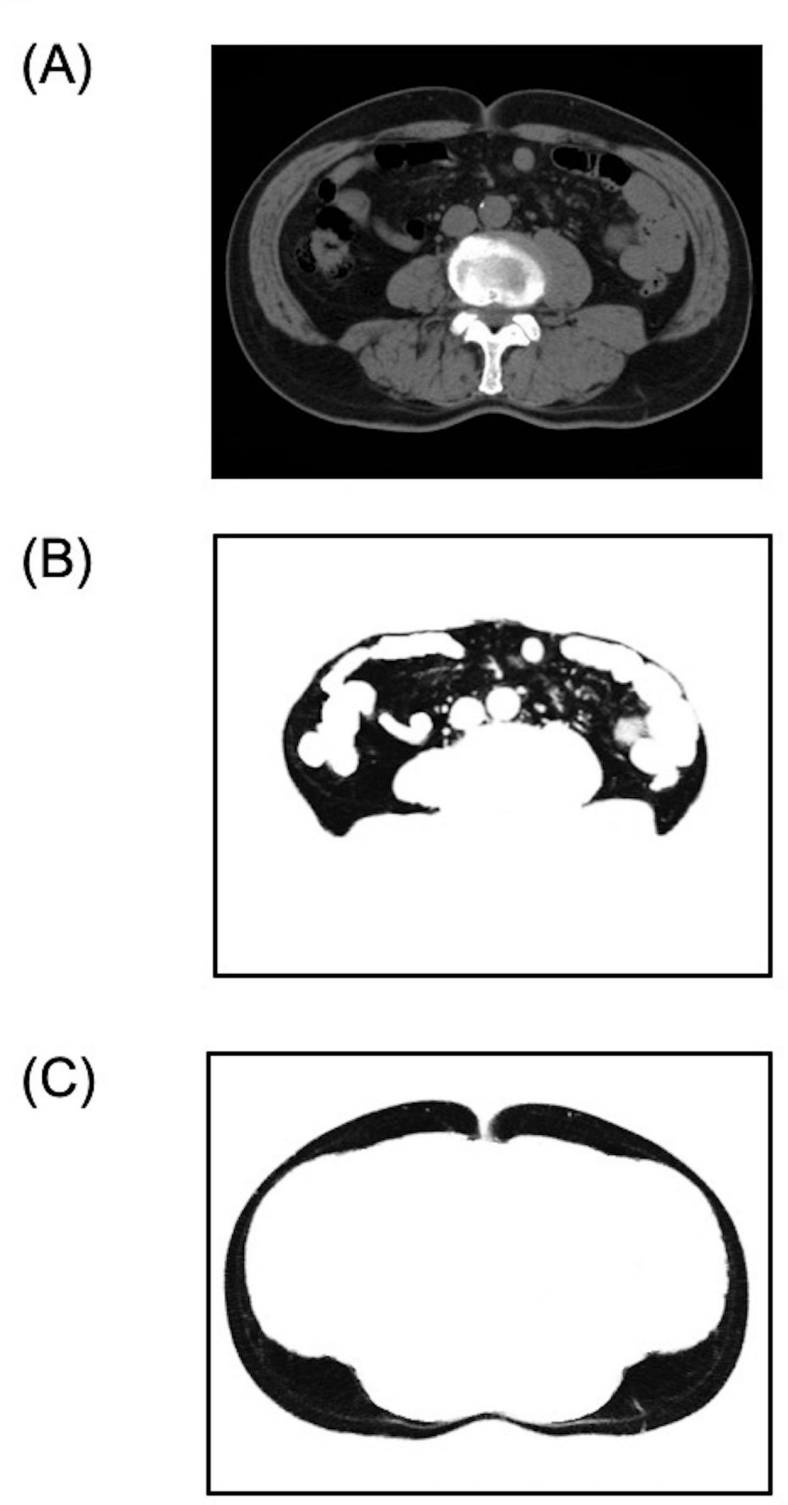
Measurements of fat using computed tomography. (A) An axial slice of a representative computed tomography image at the level of the umbilicus. (B, C) Visceral fat and subcutaneous fat after segmentation of the fat density pixels.

CT scanning was performed preoperatively in the supine position using Aquilion ONE/ViSION Edition (Canon Medical Systems Corporation, Japan). A range of -120 to -40 Hounsfield units was used to recruit fat tissue as described previously[20], and the intestinal contents were removed manually. Visceral fat was defined as the fat existing inside the region bordered by the linea alba, the abdominal muscle, and the vertebral body (Fig 1B); all fat outside this region was classified as subcutaneous adipose tissue (Fig 1C). A representative CT image is shown in Fig 1. The areas of abdominal visceral and subcutaneous adipose tissue were calculated from a CT image acquired at the level of the umbilicus using ImageJ software (National Institutes of Health, USA). The number of pixels of adipose tissue divided by the number of pixels of the total cross-sectional area was used as an index of visceral or subcutaneous fat. Two staff independently measured the CT-based amount of fat tissue, and the average of these two values was used for subsequent analyses.

**Fig 2:**
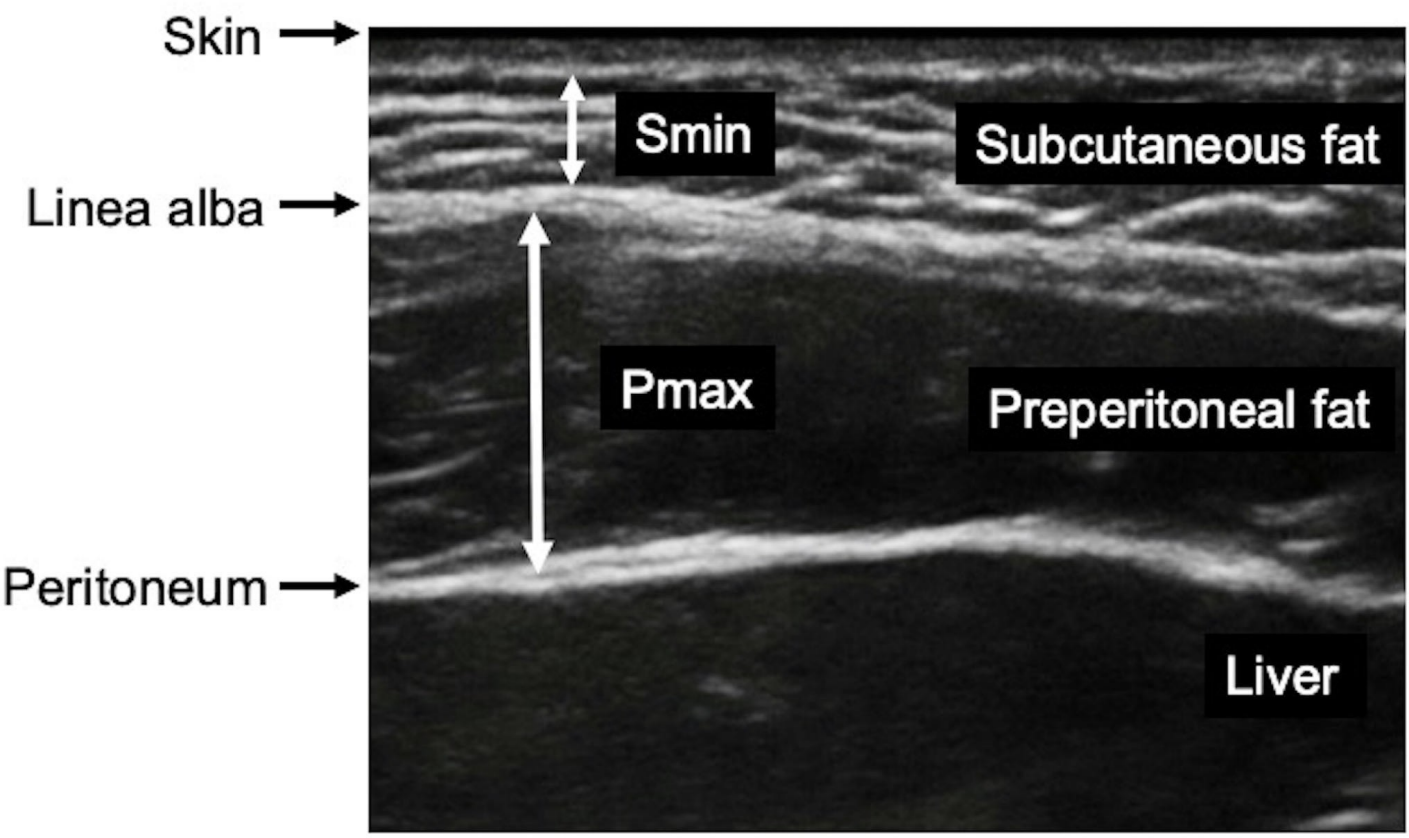
Ultrasound image of the upper abdominal wall on the xiphoumbilical line. Electronic calipers were used to measure the maximum preperitoneal fat thickness (Pmax) and minimum subcutaneous fat thickness (Smin).

An S-series ultrasound system (S-Series Ultrasound System; SonoSite, USA) was used to perform ultrasound scanning of abdominal adipose tissue using a 6–13 MHz linear transducer in accordance with Suzuki's criteria[21]. All measurements were performed during the end-expiratory phase by one of two skilled ultrasonographers, each of whom had more than 5 years of experience. We scanned longitudinally along the midline of the abdomen between the xiphoid process and the umbilicus immediately after the induction of anesthesia. The maximum thickness of preperitoneal fat and the minimum thickness of subcutaneous fat were measured as described previously[21]. We used the measured thicknesses of the preperitoneal and subcutaneous fat divided by the height as indexes of the visceral and subcutaneous fat, respectively (Fig 2).

### Statistical analysis

We conducted a multiple linear regression analysis of the change in intraoperative core temperature. Age and sex were set as fixed explanatory valuables, as these variables were considered likely to have explanatory power. The following fat-related variables were added separately as explanatory variables to avoid multiple collinearity: BMI, WHR, preperitoneal/subcutaneous fat based on ultrasonic examination, and visceral/subcutaneous fat based on CT measurements. The following variables were not associated with the intraoperative core temperature based on the Bayesian information criterion-based stepwise forward selection method, and so were not included as potential explanatory variables: doses of desflurane, remifentanil, and rocuronium, ASA-PS, bleeding volume, urine volume, time between anesthetic induction and surgical incision, initial body temperature at the time of the surgical incision, and infusion volume. All statistical analyses were conducted using JMP Pro (version 12) software (SAS Institute Inc., Cary, NC, USA). A P value of < 0.05 was considered statistically significant. Data are presented as mean ± standard deviation.

## Results

**Fig 3:**
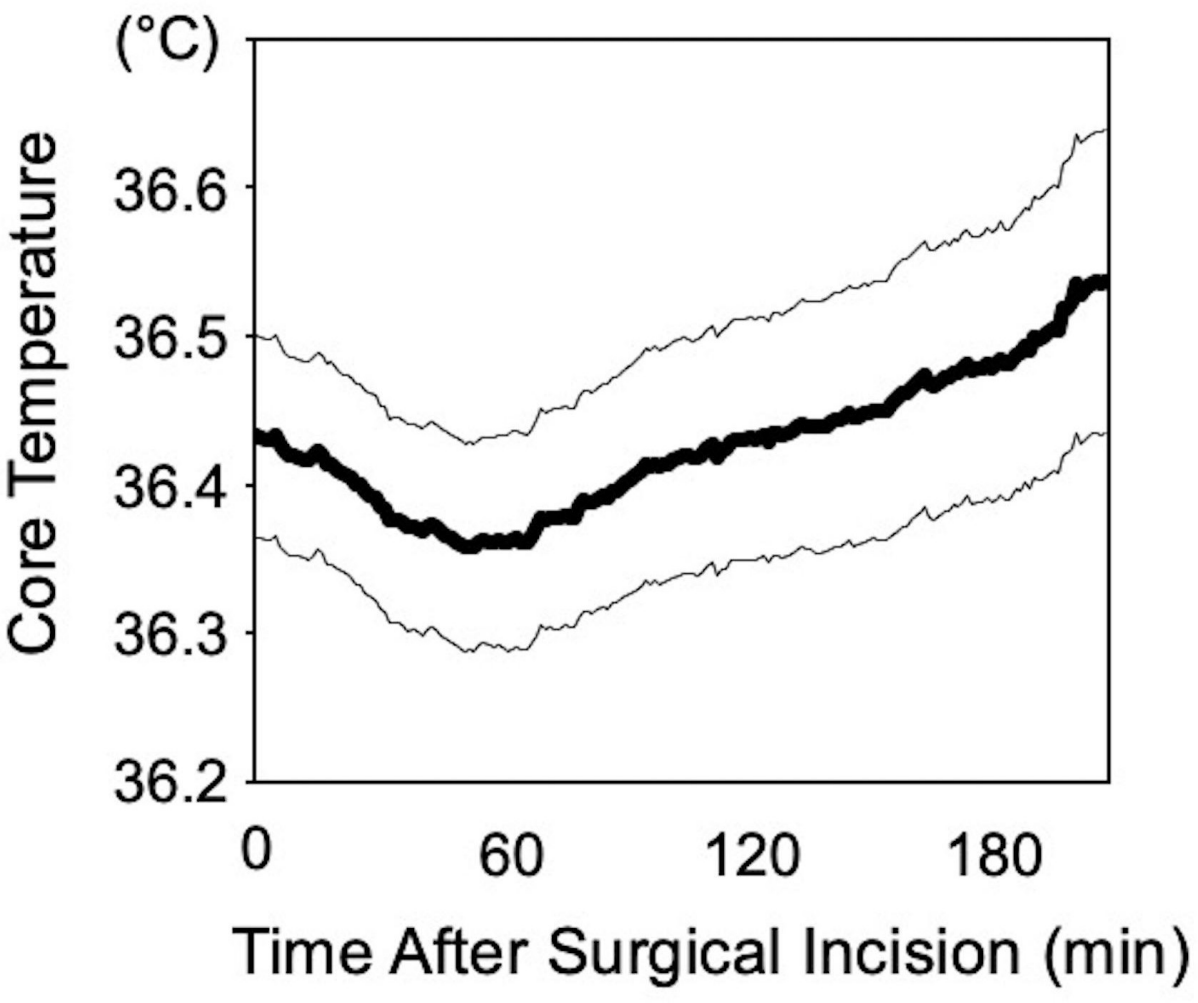
Changes in core temperature from the time of the surgical incision.

Baseline characteristics and perioperative variables of the study participants are shown in Table 1. All surgeries were completed as scheduled. All patients were classified as ASA-PS class 1 or 2. No patient required a blood transfusion, and no surgical site infections occurred. Changes in body temperature are shown in Fig 3. Core temperature decreased for about 1 hour after the surgical incision, and subsequently increased.

**Table 1:** Demographic and clinical characteristics.

Characteristic	n = 104
Age (years)	57 ± 11
Height (m)	1.61 ± 0.08
Weight (kg)	59.4 ± 11.0
BMI (kg/m^2^)	22.9 ± 3.2
Waist-to-hip ratio	0.88 ± 0.07
Sex, n (%)	
Female	68 (65%)
Male	36 (35%)
ASAPS, n (%)	
Category 1	42 (40%)
Category 2	62 (60%)
Time to incision (min)	37 ± 6
Operative time (min)	235 ± 62
Baseline body temperature (°C)	36.4 ± 0.4
Blood loss (g)	55 ± 65
Urine output (ml)	716 ± 560
Fat measurement with ultrasound	
Preperitoneal fat (mm)	8.1 ± 3.7
Preperitoneal fat/height (mm/m)	5.0 ± 2.3
Subcutaneous fat (mm)	9.6 ± 3.6
Subcutaneous fat/height (mm/m)	5.6 ± 2.2
Fat measurement with CT	
CSA (cm^2^)	474 ± 109
Visceral fat (cm^2^)	94 ± 53
Vsiceral fat/CSA (%)	19 ± 8
Subcutaneous fat (cm^2^)	150 ± 70
Subcutaneous fat/CSA (%)	31 ± 10

Time to incision: time between anesthetic induction and surgical incision. Baseline body temperature: temperature at the time of the surgical incision. Data are presented as number (%) or mean ± standard deviation. Abbreviations: BMI, body mass index; ASAPS, American Society of Anesthesiologists physical status score; CSA, cross-sectional area.

Multiple regression analysis was performed to determine the independent predictors of core temperature change. BMI was associated with core temperature change at 30 minutes after the surgical incision (Tables 2 amd 3). WHR was associated with core temperature change at 30 and 60 minutes after the surgical incision (Tables 4 and 5). Ultrasound-measured preperitoneal fat was significantly associated with increased core temperature at 30 minutes after the surgical incision, whereas subcutaneous fat was not associated with core temperature (Table 6). Similarly, CT-measured visceral fat was associated with increased core temperature at 30 minutes after the incision, whereas subcutaneous fat was not (Table 7). Ultrasound-measured preperitoneal fat was also associated with core temperature change at 60 minutes after the incision, whereas CT-measured visceral fat was not (Tables 8 and 9). Neither visceral nor preperitoneal fat were associated with core temperature at 2 hours after the surgical incision (data not shown).

**Table 2:** Multiple regression analysis (30 min after the surgical incision).

	Coefficients [95% CI]	SE	Standardized coefficients	T	P-value
Age	-0.003 [-0.006, 0.000]	0.001	-0.209	-2.22	0.029
Male	0.065 [-0.004, 0.134]	0.035	0.176	1.87	0.065
BMI	0.010 [0.001, 0.019]	0.005	0.205	2.16	0.033

Dependent variable: temperature. Abbreviations: BMI, body mass index; CI, confidence interval; SE, standard error.

**Table 3:** Multiple regression analysis (60 min after the surgical incision).

	Coefficients [95% CI]	SE	Standardized coefficients	T	P-value
Age	-0.003 [-0.007, 0.000]	0.002	-0.187	-2.00	0.049
Male	0.116 [0.030, 0.201]	0.043	0.250	2.67	0.009
BMI	0.009 [-0.002, 0.020]	0.006	0.151	1.60	0.113

Dependent variable: temperature. Abbreviations: BMI, body mass index; CI, confidence interval; SE, standard error.

**Table 4:** Multiple regression analysis (30 min after the surgical incision).

	Coefficients [95% CI]	SE	Standardized coefficients	T	P-value
Age	-0.004 [-0.007, -0.001]	0.001	-0.265	-2.65	0.009
Male	0.044 [-0.029, 0.117]	0.037	0.119	1.19	0.236
WHR	0.424 [0.065, 0.782]	0.181	0.248	2.34	0.021

Dependent variable: temperature. Abbreviations: WHR, waist-to-hip ratio; CI, confidence interval; SE, standard error.

**Table 5:** Multiple regression analysis (60 min after the surgical incision).

	Coefficients [95% CI]	SE	Standardized coefficients	T	P-value
Age	-0.004 [-0.008, -0.001]	0.002	-0.240	-2.43	0.017
Male	0.091 [0.000, 0.182]	0.046	0.197	1.99	0.049
WHR	0.462 [0.017, 0.906]	0.224	0.216	2.06	0.042

Dependent variable: temperature. Abbreviations: WHR, waist-to-hip ratio; CI, confidence interval; SE, standard error.

**Table 6:** Multiple regression analysis based on ultrasound data (30 min after the surgical incision).

	Coefficients [95% CI]	SE	Standardized coefficients	T	P-value
Age	-0.003 [-0.006, 0.000]	0.001	-0.183	-1.98	0.051
Male	0.086 [0.017, 0.156]	0.035	0.236	2.47	0.015
Preperitoneal fat/height	0.018 [0.003, 0.032]	0.007	0.234	2.42	0.018
Subcutaneous fat/height	0.015 [-0.001, 0.030]	0.008	0.188	1.90	0.060

Dependent variable: temperature. Abbreviations: CI, confidence interval; SE, standard error.

**Table 7:** Multiple regression analysis based on CT data (30 min after the surgical incision).

	Coefficients [95% CI]	SE	Standardized coefficients	T	P-value
Age	-0.003 [-0.006, 0.000]	0.002	-0.180	-1.75	0.083
Male	0.066 [-0.031, 0.163]	0.049	0.181	1.36	0.178
Visceral fat/CSA	0.005 [0.000, 0.010]	0.002	0.226	2.02	0.046
Subcutaneous fat/CSA	0.001 [-0.003, 0.006]	0.002	0.140	1.45	0.151

Dependent variable: temperature. Abbreviations: CSA, cross-sectional area; CI, confidence interval; SE, standard error.

**Table 8:** Multiple regression analysis based on ultrasound data (60 min after the surgical incision).

	Coefficients [95% CI]	SE	Standardized coefficients	T	P-value
Age	-0.003 [-0.007, 0.000]	0.002	-0.163	-1.75	0.084
Male	0.132 [0.044, 0.219]	0.044	0.287	2.97	0.004
Preperitoneal fat/height	0.019 [0.001, 0.038]	0.009	0.203	2.08	0.041
Subcutaneous fat/height	0.011 [-0.008, 0.030]	0.010	0.116	1.16	0.250

Dependent variable: temperature. Abbreviations: CI, confidence interval; SE, standard error.

**Table 9:** Multiple regression analysis based on CT data (60 min after the surgical incision).

	Coefficients [95% CI]	SE	Standardized coefficients	T	P-value
Age	-0.003 [-0.007, 0.001]	0.002	-0.162	-1.58	0.118
Male	0.110 [-0.012, 0.231]	0.061	0.239	1.79	0.076
Visceral fat/CSA	0.005 [-0.002, 0.011]	0.003	0.168	1.50	0.138
Subcutaneous fat/CSA	0.001 [-0.004, 0.006]	0.003	0.043	0.34	0.736

Dependent variable: temperature. Abbreviations: CSA, cross-sectional area; CI, confidence interval; SE, standard error.

Regression analysis showed that men tended to have a lower core temperature than women after the surgical incision. Older age was also associated with lower intraoperative core temperature.

## Discussion

The current study showed that visceral fat, but not subcutaneous fat, was strongly associated with core temperature change during laparoscopic surgery.

Visceral fat temperature is affected by the pneumoperitoneum gas temperature, whereas subcutaneous fat temperature is affected by the ambient temperature, the pneumoperitoneum gas temperature, and the effect of the forced-air warmer. The possible mechanism by which visceral fat prevents core temperature decrease is that the presence of a large amount of visceral fat minimizes the temperature decrease of internal organs or visceral fat due to pneumoperitoneum gas. Visceral fat may not influence redistribution hypothermia itself because most central heat is lost via blood-borne convection to the skin surface rather than conduction through fat tissue. Visceral fat had no explanatory power at 2 hours after the surgical incision. This may be because, at least in part, intraoperative core temperature is strongly affected by the heating effect of the forced-air warmer at several hours after induction.

We demonstrated that subcutaneous fat was not associated with core temperature change during laparoscopic surgery. However, subcutaneous fat is potentially one of the factors affecting body temperature, as subcutaneous tissue has a high insulation effect; thus, subcutaneous fat may prevent a decrease in core temperature during other kinds of surgery, or may exhibit a small heating effect of warming devices. Further research is needed to clarify whether the amount of subcutaneous fat affects the core temperature during long surgeries or during other kinds of surgery, such as head and neck surgery or limb surgery.

BMI and WHR were also predictors of core temperature at 30 minutes and 60 minutes after the surgical incision, respectively. WHR is one variable used to evaluate visceral adiposity, and is strongly associated cardiac events, whereas BMI is an indicator of heaviness rather than fatness[19, 22–25]. We therefore concluded that the reason that the explanatory power of WHR was stronger than that of BMI was because WHR is a better indicator of visceral obesity than BMI.

We chose kidney transplant donors to study the effects of visceral versus subcutaneous fat on intraoperative core temperature change for several specific reasons. To avoid possible neuropathy, no kidney transplant donors were given epidural anesthesia; thus, there was no effect of epidural anesthesia on the core temperature via peripheral vasodilation. The use of these patients also reduced other confounding factors because the donors had few comorbidities, were of a relatively young age, had relatively little perioperative bleeding, and were operated on by a single surgical team. All donor nephrectomies were performed in the renal position. Therefore, further studies are needed to evaluate core temperature changes in the supine position, as the convective heating area differs between the supine and lateral recumbent positions. However, we assume that abdominal surgery performed in the supine position would produce similar results to those in the present study, as the heating effect of the forced-air warmer is limited in the early period after the surgical incision.

The factors potentially influencing intraoperative core temperature are epidural anesthesia[26], ambient operating room temperature[10], humidity, duration between anesthesia induction and surgical incision, warming devices, and operation type. However, these factors had almost no influence on our results, as the present study was a prospective study, and all surgeries were conducted in the same operating room with the same anesthesia method by a single surgical team. In addition, blood loss and infusion volume had little effect on core temperature at the beginning of surgery.

Female sex was an independent risk factor for intraoperative core temperature decrease after adjusting for the effect of the amount of fat tissue. We considered that the reason that women had a much greater risk of core temperature decrease than men was because men have much more visceral fat than women[27]. The sex difference in the risk of intraoperative core temperature decrease may also be because men have a greater skeletal muscle mass than women[28], as skeletal muscle is an important site that produces non-shivering thermogenesis. Age was also associated with core temperature in BMI-based regression analysis, as reported in a previous study[9]. However, the present study participants were all relatively young, and so the influence of age was relatively small.

The present study had some limitations. First, our results can only be applied to patients undergoing laparoscopic surgery, and the coefficients of variables obtained from our study cannot be directly applied to other operative procedures. Second, the study participants did not receive prewarming to prevent hypothermia. Further research is needed to clarify the involvement of fat, especially subcutaneous fat, in the development of core hypothermia. Third, Asian subjects have a lower BMI than European subjects[29]. As the present patients had relatively small BMI with minimal variability, the present results may not be applicable to patients with morbid obesity. Furthermore, Asian and European subjects differ regarding fat distribution and subcutaneous/visceral fat ratio; Asian subjects have more upper-body subcutaneous fat than European subjects[30]. Further studies involving morbidly obese patients or European subjects may be necessary. Finally, we cannot discuss the effect of visceral fat on the clinical outcomes such as hypothermia or shivering, as the present patients did not experience hypothermia or associated adverse effects.

In conclusion, we demonstrated that visceral fat works protectively against core temperature decrease during laparoscopic donor nephrectomy. Further research is needed to evaluate core temperature changes during long surgeries, during other surgical procedures, and in patients who have undergone prewarming.
